# Safe and Effective Augmentation Mastopexy with Tumescent Local Anesthesia: A Decade of Experience

**DOI:** 10.3390/jcm13206057

**Published:** 2024-10-11

**Authors:** Federico Ziani, Matilde Tettamanzi, Giovanni Arrica, Roberto Cuomo, Edoardo Filigheddu, Claudia Trignano, Corrado Liperi, Corrado Rubino, Emilio Trignano

**Affiliations:** 1 Plastic Surgery Unit, University Hospital Trust of Sassari, 07100 Sassari, Italy; f.ziani@studenti.uniss.it (F.Z.); g.arrica@studenti.uniss.it (G.A.); e.filigheddu@studenti.uniss.it (E.F.); corubino@uniss.it (C.R.); etrignano@uniss.it (E.T.); 2 Unit of Plastic and Reconstructive Surgery, Department of Medicine, Surgery and Neuroscience, University of Siena, 53100 Siena, Italy; robertocuomo@outlook.com; 3 Department of Biomedical Sciences, University of Sassari, 07100 Sassari, Italy; ctrignano@uniss.it; 4 Intensive Care Unit, Emergency Department, AOU Sassari, 07100 Sassari, Italy; corrado.liperi@aouss.it; 5 Department of Medicine, Surgery and Pharmacy, University of Sassari, 07100 Sassari, Italy

**Keywords:** augmentation mastopexy, breast, tumescent local anesthesia, esthetic breast surgery

## Abstract

**Background**: Tumescent local anesthesia (TLA) is widely used in esthetic surgery due to its ability to reduce complications, eliminate the need for general anesthesia, provide effective pain control, and shorten hospitalization times. **Methods**: This study evaluates the use of TLA in 80 patients who underwent augmentation mastopexy between 2010 and 2022. A tumescent solution containing 500 mg lidocaine, 672 mg sodium bicarbonate, and 1 mg epinephrine in 1000 mL of saline was infiltrated, with an average of 300 mL per breast. The surgical technique involved creating a subpectoral pocket for textured round implants (250–400 cc), followed by careful hemostasis. **Results**: No patients required conversion to general anesthesia, and there were no signs of toxicity or major complications. Minor complications included wound dehiscence (6.2%), hematoma (2.5%), and capsular contracture (2.5%). Pain management satisfaction at 3 months post-surgery was rated as “outstanding” by 12.5% of patients, “excellent” by 67.5%, and “good” by 20%. The longest follow-up was 6 years, with no implant ruptures except one (1.2%). **Conclusions**: While the study did not include a control group or statistical analysis, the findings suggest that TLA is a safe and effective alternative to general anesthesia for augmentation mastopexy, providing excellent pain control and a low rate of complications.

## 1. Introduction

Mastopexy, commonly known as breast lift surgery, is a surgical procedure aimed at rejuvenating and reshaping the breasts by lifting and repositioning ptotic or sagging breast tissue [1]. This procedure is sought by individuals seeking to restore a more youthful breast contour and address concerns related to breast ptosis, which can arise due to factors such as aging, pregnancy, breastfeeding, or significant weight loss [2]. While mastopexy is recognized for its ability to enhance breast esthetics and boost self-confidence [3], advancements in surgical techniques and anesthesia protocols continue to refine the procedure, with the goal of optimizing outcomes and patient satisfaction [4]. Tumescent local anesthesia has emerged as a valuable adjunct in mastopexy surgery, offering several potential benefits including improved pain control, reduced intraoperative bleeding, and enhanced recovery after surgery (ERAS) [5,6,7]. This anesthesia technique involves the infiltration of a dilute solution containing local anesthetics such as lidocaine [8,9], and vasoconstrictors such as epinephrine, into the breast tissue and surrounding areas. However, it is important to note that while TLA supports ERAS protocols, it does not replace the need for an anesthesiologist. An anesthesiologist remains essential for patient monitoring and managing any complications during the procedure [10]. By achieving anesthesia and vasoconstriction, tumescent local anesthesia facilitates precise surgical dissection while minimizing blood loss and postoperative discomfort [10,11]. Despite the increasing adoption of tumescent local anesthesia in mastopexy procedures [7], comprehensive scientific investigations evaluating its efficacy, safety profile, and perioperative outcomes are relatively limited. Previous studies, such as Zucal et al. [12], have highlighted the complexity of augmentation mastopexy and the need for the further evaluation of outcomes to better inform surgical protocols. Therefore, this study sought to address this gap by systematically examining the clinical outcomes and patient-reported experiences associated with mastopexy performed under tumescent local anesthesia. Additionally, our study aimed to expand the existing case series to facilitate future comparative studies and further contribute to the body of evidence on the safety and efficacy of this anesthesia technique. By elucidating the advantages and potential limitations of tumescent local anesthesia in mastopexy surgery, this research aimed to contribute to the refinement of surgical techniques and perioperative care protocols. Through meticulous analysis of surgical outcomes, complication rates, and patient satisfaction measures, this study endeavored to provide valuable insights that can inform clinical practice and further enhance the management of breast ptosis.

## 2. Materials and Methods

Between 2010 and 2022, a total of 80 patients underwent bilateral mastopexy with augmentation. All surgeries were conducted in an accredited outpatient clinic. The surgical team consisted of a board-certified plastic surgeon, an assistant surgeon, an operating room nurse, and a board-certified anesthesiologist, whose presence was crucial for monitoring the patient and ensuring safety throughout the procedure. The patients were thoroughly informed about mastopexy with augmentation, including the indications and potential complications (such as implant infections and postoperative bleeding). Preoperative assessments included routine blood tests, an electrocardiogram (ECG) with a cardiology consultation, and breast imaging via ultrasound and/or mammography. Mammography was indicated following ultrasound in cases where further clarification of suspicious findings was required. All medications affecting the coagulation system were discontinued in accordance with international guidelines. All patients met the criteria of the American Society of Anesthesiologists (ASA) for status I or II. Exclusion criteria were ASA status III or more, pregnancy, BMI over 35, and BI-RADS classification 4 or higher. Patients with a BI-RADS score of 3 or lower were included, with BI-RADS 3 patients required to undergo a 6-month follow-up to monitor potential changes [13]. The breast implants used had a silicone gel content, featured a textured silicone surface, and had a round shape (Brands included Nagor, Politech, Mentor, and Motiva). Implant sizes were determined based on the breast diameters, the dimensions of the anterior thoracic wall, and the weight of the breast tissue excised during surgery. Preoperative markings were made with the patient in an upright position, and photographs were taken prior to administering anesthesia. Peripheral intravenous access was placed for each patient, and vital signs were continuously monitored throughout the surgery and recovery period. Tumescent solution was prepared with 500 mg of lidocaine, 672 mg of sodium bicarbonate, and 1 mg of epinephrine in 1000 mL of 0.9% saline solution. As a reminder, the maximum recommended dose of lidocaine is 4.5 mg/kg without epinephrine and 7 mg/kg when combined with epinephrine [14]. However, the safe dose of lidocaine for TLA is much higher, with recommendations between 28 and 55 mg/kg [15]. These limits were strictly observed throughout the procedure to ensure patient safety. Overall, 250–350 mL was introduced per breast. The surgical incision site was infiltrated with 1% lidocaine and 1:100,000 epinephrine. During the anesthesia phase, the plane between the gland and the superficial fascia of the pectoralis major muscle was identified by gently pinching the breast against the chest wall. A spinal needle, connected to a peristaltic infiltration pump, was then inserted into this plane. The device was stopped once the glands became turgid and vasoconstricted; in our case series, this corresponded to a mean volume of 300 mL per breast. The volume of tumescent solution infiltrated varied based on breast size and the patient’s BMI. For patients with smaller breasts and lower body weight, a smaller volume of tumescent solution was required to achieve breast turgidity and avoid reaching toxic levels of the drug. The initial incision was made 20 min after infiltration to ensure the full effect of both epinephrine and lidocaine. We preferred to wait 20 min to ensure a more effective hemostatic effect and to maximize the anesthetic efficacy, allowing for optimal vasoconstriction and minimizing intraoperative bleeding before making the incision.

### 2.1. Surgical Technique

The incisions were made according to the preoperative markings. A 45 mm diameter circle was drawn around the nipple–areola complex (NAC) and incised. The modified Wise pattern was deepithelialized, with careful attention to preserving the NAC. The apex of the inferior flap was incised at the base of the medial and lateral pillars, aligning with the base of the Wise pattern. Incisions were then extended from the edges of the inferior flap down to the muscle, starting below the NAC pedicle. The inferolateral border of the pectoralis major muscle was identified by dissecting the breast parenchyma just lateral to the inferior pedicle. After exposing the fascia of the pectoralis major muscle, 1 mL of 1% lidocaine with 1:100,000 epinephrine was injected, and a blunt, multi-fenestrated 2 mm cannula was inserted and secured within the muscle using a single 4–0 silk round block suture. Following this, between 160 and 220 mL of tumescent solution was injected using a Luer-lock syringe. Once infiltration was complete, a subpectoral pocket was created to accommodate the implant. The size of the implants ranged from 250 cc to 400 cc, selected based on breast dimensions, patient preference, and the amount of tissue removed during the procedure. The progressive coagulation of blood vessels was carried out during the dissection of the pocket, prior to implant insertion, to prevent secondary bleeding following the effect of vasoconstriction. A fiberoptic retractor with smoke evacuation capabilities was utilized during the dissection. Before implant placement, sterile drapes and gloves were replaced, and the pocket was irrigated sequentially with a 50% diluted hydrogen peroxide solution, saline solution, and a gentamicin solution. Surgical drains were not employed. The wound was closed in layers using absorbable sutures, and a sterile dressing was applied.

### 2.2. Postoperative Management

**Figure 1:**
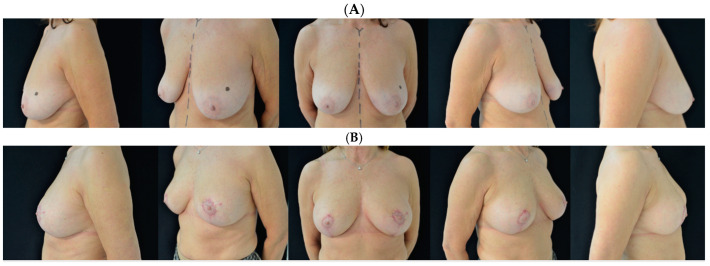
A 63-year-old woman is shown (**A**) before and (**B**) at 6 months follow-up.

**Figure 2:**
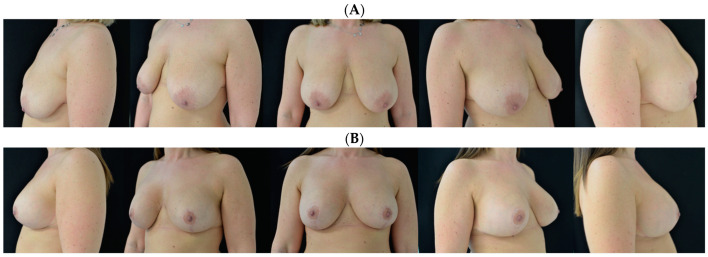
A 50-year-old woman is shown (**A**) before and (**B**) at 1 year follow-up.

**Figure 3:**
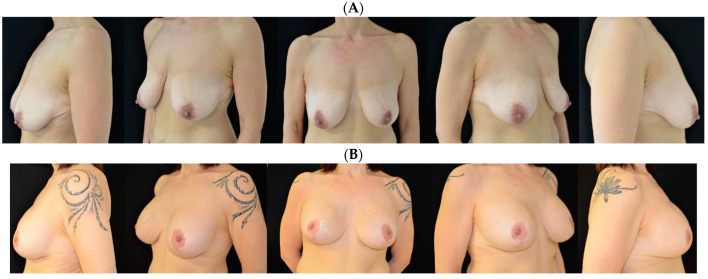
A 38-year-old woman is shown (**A**) before and (**B**) at 3 years follow up.

Following surgery, patients were instructed to wear a supportive sport bra for 1 month. After 4 h of observation, they were discharged. Based on allergy status, an oral antibiotic (either amoxicillin 875 mg/clavulanic acid 125 mg or ciprofloxacin 500 mg, taken twice daily) was prescribed for 5 days. Postoperative follow-up visits were scheduled at 1 day, 1–2 weeks, 1 month, 3 months, 6 months, and 1 year (Figure 1, Figure 2 and Figure 3). Patient satisfaction was evaluated using a satisfaction survey conducted 3 months post surgery. In this survey, patients were asked to rate their pain management and satisfaction with the esthetic results on a scale from “unsatisfactory” to “excellent”.

## 3. Results

A total of 80 patients underwent surgery during the study period and were subsequently included in this retrospective analysis. Patients mean age was 42 years (range, 24–67 years), while the mean body weight was 64 kg (range, 55–71 kg) and the mean Body Mass Index (BMI) was 27.1 kg/m^2^ (range, 24.6–28.7 kg/m^2^). No major complications were observed, and no conversions to general anesthesia were necessary. Additionally, there were no reports of epinephrine or lidocaine toxicity, nor were there any electrocardiographic changes, respiratory depression, or incidents of acute hypotension or hypertension. No patients exhibited symptoms of hypothermia (such as slurred speech, shallow breathing, weak pulse, clumsiness, drowsiness, confusion, memory loss, loss of consciousness, or bright red, cold skin), with the exception of mild shivering that lasted, on average, for 15 min immediately after surgery. The median surgery time was 70 min (range, 60–80 min), and all patients were discharged within 2 h after surgery. Table 1 shows the minor complication rate at the 1-year follow-up. Wound dehiscence was observed in five cases (6.2%), and hematoma in two patients (2.5%). No wound or implant infections were observed and postoperative bleeding requiring a return to theater was never necessary. In two cases (2.5%), patients developed capsular contracture, and one patient experienced a rupture of the implant (1.2%) within the follow-up time (1 year). Patients expressed satisfaction with the TLA procedure, reporting no discomfort during the preoperative infiltration or throughout the entire surgical process. Most patients reported high satisfaction at the survey conducted 3 months after surgery with the esthetic results, with the majority rating their experience as “satisfactory” to “excellent” (Table 2). A small percentage expressed low level of satisfaction, primarily those who experienced complications such as implant dislocation, which led to corrective surgery to improve esthetic outcomes. Additionally, the majority rated their postoperative pain management as “excellent” (Table 3). All patients were followed for more than 1 year, with 42 patients having a follow-up period of 6 years, the longest in our experience with this technique. The shortest follow-up in this case series was 1 year, which applied to six patients.

## 4. Discussion

In this article, we present our 12-year experience with 80 cases of augmentation mastopexy performed using tumescent local anesthesia (TLA). Our postoperative complication rate was 16.2%, including hematoma (2.5%), dystrophic scars (6.2%), implant dislocation (3.7%), and seroma formation (3.7%), which are commons breast implant surgery complications [16,17]. Notably, three reinterventions (3.7%) were required to correct the cases of implant dislocation. Conversion to general anesthesia was never necessary, and no adverse events during TLA were recorded. Over the years, various authors have proposed different local anesthesia protocols for breast surgeries. Tumescent local anesthesia (TLA) has proven effective for esthetic surgeries, especially for breast augmentation [10,11,18,19,20], intramuscular gluteal augmentation [21,22], abdominoplasties [23], and arm lifting [24]. This technique was pioneered by Klein [25] for liposuction and has evolved over time, seeing widespread use due to its lower complication rates, elimination of the need for general anesthesia, reduced pain, decreased use of narcotics, and shorter recovery time.

Similarly, other local and regional anesthesia techniques have been explored in various surgical contexts, contributing to the growing trend in minimizing general anesthesia [26,27,28,29]. It has been observed that in regions with a high prevalence of autoimmune neuromuscular disorders such as Myasthenia Gravis, the use of tumescent local anesthesia (TLA) instead of general anesthesia may be preferable [21]. These patients are particularly sensitive to the respiratory depressant effects of general anesthesia and muscle relaxants. Since pain and stress can worsen symptoms of myasthenia gravis, TLA can help reduce postoperative complications in this patient population. Despite this, many surgeons continue to perform augmentation mastopexy under general anesthesia and, although local anesthesia protocols are gradually gaining popularity, there are still limited studies in the literature on the use of local anesthesia for augmentation mastopexy, making it difficult to establish a comprehensive basis for comparison. A study by Alex Colque et al. [30] describes the use of intercostal nerve blocks and intravenous sedation. They performed an intercostal nerve block with a local anesthesia solution, consisting of equal parts of 0.25% bupivacaine and 1% lidocaine with 1:100,000 epinephrine, in an area extending from Intercostal Spaces 3–7 at the midaxillary line and at the lateral sternal border to provide a lateral and medial block to the breast. They also injected the solution into the operating field during dissection. The sedation was administered by the surgeon by injecting intravenously 1 mg of midazolam. Additionally, for analgesia, 50 μg of fentanyl and 10 mg of ketamine were administered. Although augmentation mastopexy procedures usually require a longer operating time compared to augmentation alone, in the study, they managed to avoid prolonging the recovery room stay. This success is likely due to the effectiveness of the intercostal nerve block for postoperative pain control. On the other hand, almost 13% of patients experienced postoperative nausea, likely due to the use of ketamine and fentanyl [31,32]. However, some studies reported that intravenous anesthesia protocols have been shown to cause a lower incidence of these side effects compared to general anesthesia [33,34]. Our protocol’s data demonstrate that in avoiding the use of propofol, ketamine, and fentanyl, we reduce the perioperative risk caused by these drugs such as respiratory depression, blood pressure fluctuations, bradycardia, nausea, and vomiting. In accordance with the literature [35], we highly advise having an anesthesiologist present throughout the procedure to monitor oxygen saturation continuously and evaluate the patient’s respiratory and cardiocirculatory status. Moreover, it is recommended to perform these procedures in facilities equipped for immediate conversion to general anesthesia. In our protocol, we exclusively employed tumescent local anesthesia (TLA), eliminating the necessity for intravenous anesthesia or nerve blocks. This strategy offers substantially lower risks, as nerve blocks may occasionally lead to block failure, bleeding, hematoma, or neurological injury, potentially requiring a switch to general anesthesia [36]. Tumescent anesthesia effectively minimizes bleeding and enables efficient work in both the subglandular and sub-muscular planes. Furthermore, nerve blocks mandate the use of an ultrasound probe and individuals with specialized skills, increasing procedural costs. In an approach similar to ours, Ceccarino et al. [7] already proposed a protocol for augmentation mastopexy with cold tumescent anesthesia (CTA). In this protocol, a solution at lower temperature (4 °C) is injected, and an intravenous sedation through midazolam is administered. Our data did not show significant differences in terms of complications compared to those reported by the author. About pain control, in a study by Joukhadar et al., it was reported that refrigerated local anesthetics cause more pain upon injection compared to room-temperature solutions [37,38]; therefore, cold tumescent solution increases patient discomfort due to chills caused by the drop in temperature and increased pain during the injection. We believe that although temperature may affect the drug’s onset, it is still necessary to wait a minimum amount of twenty minutes to ensure the best possible analgesia for the patient. Indeed, in our experience, patients did not experienced pain during the injection with a room temperature solution, but rather an increased sense of pressure. In the literature there are no comparative studies that have documented any advantages on chilled tumescent injection over room temperature injections; on the other hand, it is well known that cold solution injections may cause hypothermia, and for this reason, the patient’s temperature should always be constantly monitored. This condition could lead to cardiac events such as ventricular fibrillation [39,40], and, in some cases, it has been associated with increased surgical bleeding, probably because platelet function and the coagulation cascade are impaired by hypothermia, as demonstrated in in vitro studies [41,42,43]. Similarly to the author, we selectively chose patients classified as ASA I or II based on the American Society of Anesthesiologists (ASA). In our protocol, the procedure is conducted under local anesthesia without the administration of midazolam, thereby decreasing the risk of patient dissociation and aiding in shortening the recovery time. Strictly adhering to these criteria is particularly crucial for ensuring the safety and efficacy of the procedure. When utilizing our technique, we recommend performing the surgical steps with caution, as changes to the surgical plan cannot be made intraoperatively. The infiltration should be conducted in a controlled environment with careful monitoring to ensure that the correct volume of solution is used. It must be administered gradually, with the surgeon palpating the breast to confirm that the solution is flowing into the proper plane. Sub-muscular implant positioning requires the precise sectioning of the pectoralis major muscle insertions at the level of the ribs and sternum. This step is crucial yet can lead to bleeding due to the severing of the perforating branches of the internal thoracic artery and vein [11,44]. To mitigate this, tumescent local anesthesia (TLA) containing epinephrine is administered, inducing vasoconstriction that helps minimize blood loss and bleeding throughout the surgical procedure [45]. The dissection of the pocket for implant placement is executed using a combination of blunt dissection and cautery. However, the presence of a substantial amount of fluid can impede cautery dissection. Therefore, it is imperative for the surgeon’s assistant to continuously apply suction to the surgical site to remove excess fluid. This facilitates easier cautery dissection, enhancing surgical precision. Despite the absence of muscle relaxants, maintaining muscular tone is not a significant concern during the surgery. This comprehensive approach ensures effective control of bleeding, optimal visibility, and successful sub-muscular implant positioning. If hemostasis is effectively achieved, drains may not be necessary, thus reducing patient discomfort and minimizing the risk of implant infection. Indeed, we observed hematoma occurrence in only two patients (2.5%) and seroma formation in three patients (3.7%). Due to the potential impact of the injected solution volume on breast shape, precise preoperative markings are crucial, as intraoperative adjustments are not feasible. Patients should be informed that swelling of the breasts is expected during the initial postoperative weeks. We documented only three cases of implant dislocation. Another minor complication we encountered was dystrophic scarring and delayed wound closure. In such instances, we applied a polyurethane dressing to aid wound closure [11,46]. In our experience with augmentation mastopexy using TLA, we obtained fully satisfied patients. Comprehensive postoperative care, including the close monitoring of tissue perfusion and wound healing, is essential in preventing complications and ensuring optimal surgical outcomes [47]. Even though this procedure is invasive due to the creation of a sub-muscular pocket, which can cause significant intraoperative and postoperative pain, we can effectively manage it using TLA and prescribing mild analgesics for the postoperative period. The pain level was assessed by the anesthesiologist during the procedure. After surgery and during follow-up, patients were asked to rate pain management by giving a score from “satisfactory’’ to ‘‘excellent” with no complaints. As we reported in a previews study [11], during the intraoperative pain management, most of the patients suffered almost complete amnesia caused by midazolam [48]; we believe that, for this reason, it is crucial for the anesthesiologist to conduct the pain assessment at this stage. Moreover, thanks to TLA, we can reduce the risk of DVP (Deep Venous Thrombosis) thanks to the early mobilization of the patient and the early discharge [11,49,50]. After describing our methodology and findings, it is important to acknowledge certain limitations in this study. The relatively small sample size and the lack of a control group limit the extent to which these findings can be generalized. Additionally, the single-center design with all procedures conducted by the same surgical team may introduce bias, and this study’s retrospective nature also brings inherent limitations.

## 5. Conclusions

While our study demonstrates that tumescent local anesthesia (TLA) is a safe and effective option for augmentation mastopexy, with high patient satisfaction and low complication rates, several limitations must be acknowledged. The small sample size limits the generalizability of our findings, and the absence of both a control group and formal statistical analysis reduces the strength of our conclusions, which are primarily based on the authors’ clinical experience. Future studies should include larger, multi-center cohorts with control groups and prospective data collection to provide more definitive insights into TLA’s long-term impact. Additionally, examining different patient populations, including those with higher ASA scores or comorbidities, could extend the applicability of TLA for broader surgical use. Exploring these aspects would contribute valuable information to the growing body of evidence on TLA. Despite these limitations, TLA offers significant potential for specific patient populations, particularly those who may not be ideal candidates for general anesthesia, such as older patients, individuals with comorbidities, or those seeking outpatient procedures with faster recovery times. Based on our experience, we recommend that surgeons employing TLA in augmentation mastopexy ensure careful preoperative planning and patient selection. It is essential to combine this anesthesia technique with proper surgical methods and meticulous hemostasis to prevent complications such as hematoma. Additionally, any clinic using tumescent local anesthesia should be equipped with appropriate safety measures, including the availability of intravenous lipid emulsion therapy for the management of potential local anesthetic systemic toxicity (LAST). We also strongly recommend the continuous presence of a board-certified anesthesiologist in the operating room to monitor the patient’s respiratory and circulatory status throughout the procedure. These precautions are essential for ensuring patient safety and effectively managing any anesthetic emergencies. In conclusion, while further research is required, TLA presents a promising alternative to traditional anesthesia approaches in augmentation mastopexy, offering benefits in terms of reduced recovery times, improved patient satisfaction, and lower perioperative risks in selected patient groups.

## Figures and Tables

**Table 1:** Patients’ minor complications.

Complications	Patients	%
Hematoma	2	2.5%
Seroma	3	3.7%
Implant dislocation	3	3.7%
Dystrophic scars	5	6.2%
Need for reintervention	3	3.7%

**Table 2:** Esthetic satisfaction survey at 3 months follow-up.

Scale	Patients	%
Outstanding	18	22.5%
Excellent	47	58.75%
Good	14	17.5%
Satisfactory	1	1.25%
Unsatisfactory	0	0

**Table 3:** Pain management survey at 3 months follow-up.

Scale	Patients	%
Outstanding	10	12.5%
Excellent	54	67.5%
Good	16	20%
Satisfactory	0	0%
Unsatisfactory	0	0

## Data Availability

Databases can be provided upon request.
